# Repetitive Transcranial Magnetic Stimulation in Cervical Dystonia: Effect of Site and Repetition in a Randomized Pilot Trial

**DOI:** 10.1371/journal.pone.0124937

**Published:** 2015-04-29

**Authors:** Sarah Pirio Richardson, Sule Tinaz, Robert Chen

**Affiliations:** 1 Department of Neurology, University of New Mexico Health Sciences Center, Albuquerque, New Mexico, United States of America; 2 Human Motor Control Section, National Institute of Neurological Disorders and Stroke, National Institutes of Health, Bethesda, Maryland, United States of America; 3 Toronto Western Research Institute, University of Toronto, Toronto, Canada; University of Regensburg, GERMANY

## Abstract

Dystonia is characterized by abnormal posturing due to sustained muscle contraction, which leads to pain and significant disability. New therapeutic targets are needed in this disorder. The objective of this randomized, sham-controlled, blinded exploratory study is to identify a specific motor system target for non-invasive neuromodulation and to evaluate this target in terms of safety and tolerability in the cervical dystonia (CD) population. Eight CD subjects were given 15-minute sessions of low-frequency (0.2 Hz) repetitive transcranial magnetic stimulation (rTMS) over the primary motor cortex (MC), dorsal premotor cortex (dPM), supplementary motor area (SMA), anterior cingulate cortex (ACC) and a sham condition with each session separated by at least two days. The Toronto Western Spasmodic Torticollis Rating Scale (TWSTRS) score was rated in a blinded fashion immediately pre- and post-intervention. Secondary outcomes included physiology and tolerability ratings. The mean change in TWSTRS severity score by site was 0.25 ± 1.7 (ACC), -2.9 ± 3.4 (dPM), -3.0 ± 4.8 (MC), -0.5 ± 1.1 (SHAM), and -1.5 ± 3.2 (SMA) with *negative* numbers indicating improvement in symptom control. TWSTRS scores decreased from Session 1 (15.1 ± 5.1) to Session 5 (11.0 ± 7.6). The treatment was tolerable and safe. Physiology data were acquired on 6 of 8 subjects and showed no change over time. These results suggest rTMS can modulate CD symptoms. Both dPM and MC are areas to be targeted in further rTMS studies. The improvement in TWSTRS scores over time with multiple rTMS sessions deserves further evaluation.

**Trial Registration:**

ClinicalTrials.gov NCT01859247

## Introduction

After Parkinson disease and essential tremor, dystonia is the third most common movement disorder. It is characterized by abnormal posturing due to sustained muscle contractions, which not only leads to pain but also often causes significant disability in activities of daily living such as driving and reading. Primary focal dystonia such as cervical dystonia (CD) affects both genders and all ethnicities and races. The current gold standard treatment of botulinum toxin injections has limitations—painful, frequent injections as well as expected adverse events, which can include dysphagia, neck weakness, voice changes and fatigue [1]. In addition, many CD patients do not have relief of their symptoms for the entire treatment period as the effect “wears off” prior to the next injection. Employment is affected by CD and in one study, a large percentage of patients were not employed even with standard treatment [2]. New therapies and new therapeutic targets are urgently needed in this disorder.

Previous work implies that CD is a *neurofunctional* disorder arising from abnormalities of neural connectivity or plasticity, rather than being caused by neurodegeneration [3]. The functional rather than structural nature of the disorder raises the possibility that symptoms of CD can be persistently modified through noninvasive means. Several trials using low-frequency repetitive transcranial magnetic stimulation (rTMS) over the premotor cortex have shown modulation of symptoms in focal hand dystonia (FHD) [4–7]. In one pilot study, patients with secondary dystonia received low-frequency rTMS over the premotor cortex and showed significant improvement in painful spasms in proximal and axial musculature [8]. A subsequent case report demonstrated a 50% reduction in CD symptoms in a patient who had received a course of low-frequency rTMS over the premotor cortex, and the effects were sustained for four months [9]. Our previous work has also identified an abnormal premotor-motor interaction in patients with focal dystonia in both writer’s cramp [10,11] and CD [12]. Thus, the premotor cortex may be an appropriate target for therapeutic intervention. The motor cortex (MC) has also been evaluated as a therapeutic site. Low-frequency rTMS over the MC in a single session showed a decrease in writing pressure in writer’s cramp patients [13].

Given these results in the FHD population, we proposed this randomized, sham-controlled, blinded exploratory pilot study to identify a specific motor system target and to evaluate this target in terms of safety and tolerability in the CD population.

## Materials and Methods

### Participants

**Fig 1:**
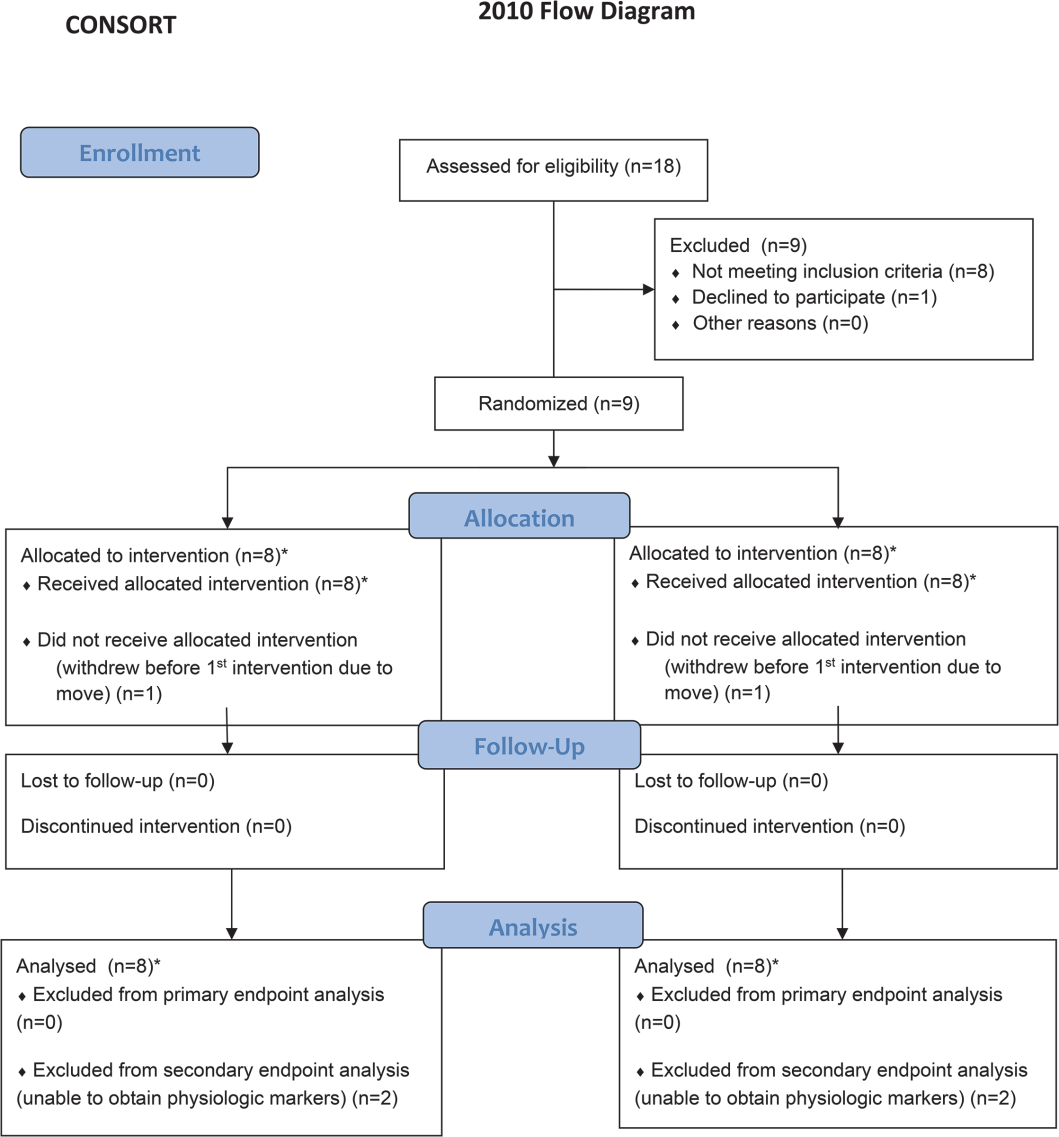
Consort diagram. *Prior to enrolment, a randomization schedule was created by random sorting of the five rTMS sites for each subject. All 8 subjects each received rTMS to five separate sites including sham over the course of the trial. (See Table 2 for the randomized order of interventions by subjects).

The protocol for this trial and supporting CONSORT checklist are available as supporting information; see S1 CONSORT Checklist and S1 Protocol. We enrolled 9 CD patients (5 men) with mean age 53 years (standard deviation (SD) 16) (Table 1). As there was no prior data on the influence of rTMS on patients with CD using our primary outcome measure, we were not able to calculate a formal sample size and thus estimated that enrolling approximately 7 patients would be a reasonable approach for an exploratory trial. Patient recruitment began June 1, 2013 and patient follow-up was completed by July 31, 2014. Eight CD patients completed the study. One patient withdrew after enrolling, but prior to beginning any study intervention, due to an unexpected move out of state (Fig 1). The study intervention began at 8 weeks after their usual care botulinum toxin injection. All subjects met inclusion criteria: 1) patients with idiopathic cervical dystonia; 2) age 18 years or older; 3) normal findings in the medical history, physical and neurological examination, except for dystonia; and 4) last treatment with botulinum toxin more than two months prior to first intervention. All subjects did not have any exclusion criteria: 1) history of seizure disorder; 2) pregnancy- a pregnancy test will be performed for women of childbearing potential; 3) symptoms of a clinically relevant illness in the 4 weeks before the first study day, including history of any other neurological disorders or conditions requiring the use of anti-depressants that are known to increase seizure threshold, neuroleptic medication, anticholinergic drugs and muscle relaxants with the exception of benzodiazepines; 4) history of neuroleptic medications/ prior use of neuroleptics; and 5) presence of pacemaker, implanted medical pump, metal plate or metal object in skull or eye. They all signed an informed consent. The study was approved by the University of New Mexico Health Sciences Center Institutional Review Board.

**Table 1:** Demographic, clinical features and baseline severity by participant and by group.

SUBJECT	AGE^	SEX	DISEASE DURATION^	BASELINE TWSTRS			
				I. SEVERITY	II. DISABILITY	III. PAIN	TOTAL
1	33	M	2	20	12	4.25	36.25
CLINICAL FEATURES: LEFT TURN, RIGHT TILT, RIGHT SHOULDER ELEVATION							
2	60	M	7	9	9	7.75	25.75
CLINICAL FEATURES: ANTEROCOLLIS (DOUBLE CHIN)							
3	67	F	16	8	8	0	16
CLINICAL FEATURES: RETROCOLLIS WITH DYSTONIC TREMOR							
4	31	M	4	14	0	0	14
CLINICAL FEATURES: RIGHT TURN, RIGHT SHOULDER ELEVATION, LEFT LATERAL SHIFT							
5	55	F	7	21	4	15.5	57.5
CLINICAL FEATURES: LEFT TURN, RETROCOLLIS, DYSTONIC TREMOR							
6	41	M	7	20	13	13.75	46.75
CLINICAL FEATURES: RIGHT TURN, RIGHT SHOULDER ELEVATION							
7	65	F	20	12	4	6.25	22.25
CLINICAL FEATURES: RIGHT TURN, RETROCOLLIS, DYSTONIC TREMOR							
8	72	M	4	16	22	12	50
CLINICAL FEATURES: ANTEROCOLLIS							
GROUP	53 (16)		8.3 (6.3)	15 (5.1)	9 (6.8)	7.4 (6)	33.6 (16.5)

^^^ in years

**Table 2:** Demographic, clinical features and baseline severity, and raw TWSTRS severity scores by participant.

SUBJECT	INTERVENTION									
	1		2		3		4		5	
	PRE	POST	PRE	POST	PRE	POST	PRE	POST	PRE	POST
1	MC		SMA		SHAM		dPM		ACC	
	20	20	20	17	19	16	9	3	3	3
2	ACC		MC		dPM		SHAM		SMA	
	9	9	9	7	7	5	5	5	5	5
3	dPM		SMA		SHAM		MC		ACC	
	8	8	8	8	8	8	8	8	4	4
4	SMA		SHAM		ACC		MC		dPM	
	14	14	14	14	14	14	13	4	14	5
5	SHAM		dPM		MC		SMA		ACC	
	21	21	21	21	21	21	21	21	21	21
6	ACC		dPM		SMA		SHAM		MC	
	20	18	20	20	20	20	20	20	20	20
7	SHAM		MC		dPM		ACC		SMA	
	12	12	12	11	12	11	7	7	7	7
8	ACC		SMA		MC		SHAM		dPM	
	16	20	17	8	20	8	21	20	21	16
GROUP	15 (5)	15.3 (5.2)	15.1 (5.1)	13.3 (5.6)	15.1 (5.7)	12.9 (5.7)	13 (6.7)	11 (7.9)	11.9 (8)	10.1 (7.6)

### Study Design

#### Study Events

**Fig 2:**
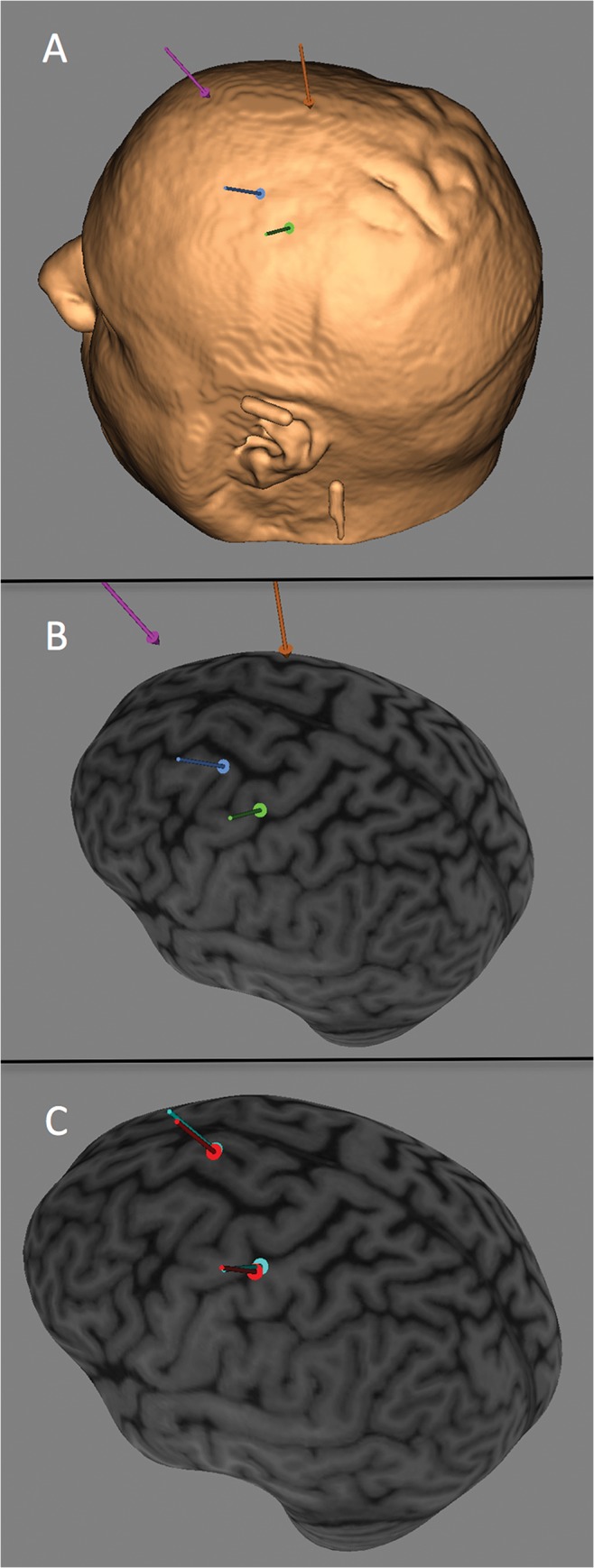
Scalp and cortical reconstructions. A) Scalp reconstruction of subject showing scalp locations of TMS hotspot targets by site (green = MC, blue = dPM, orange = SMA, pink = ACC). B)Cortical surface reconstruction of same subject showing same locations targeted (note that the arrows are suspended above the brain as they are marking the scalp locations of stimulation). C)Cortical surface reconstruction showing session to session consistency over targets (light blue = session 1 and red = session 3).

A non-contrasted anatomical brain magnetic resonance imaging (MRI) was performed for each subject prior to the first study day for registration in the neuronavigation system. The rTMS interventions were guided by a neuronavigation system (Brainsight) to ensure consistent placement of coil from session to session (Fig 2).

At the beginning of each session, participants were seated in a comfortable chair in the Noninvasive Neurostimulation Lab at the Unviersity of New Mexico and underwent a standardized, scripted videotaping for use by the blinded reviewer in rating their TWSTRS severity score. After videotaping, participants had electrodes applied over the right first dorsal interosseous (FDI) muscle as described below. Earplugs and cap were placed. Registration with the neuronavigation system was completed and then the hotspots were identified (as described below) for the MC and dPM. These locations were saved in the neuronavigation system for future reference. Depending on the randomization schedule, additional anatomical sites were determined. Neurophysiologic testing (dorsal premotor-motor cortical inhibition (dPMI) and cortical silent period (CSP)) was performed. The rTMS session was completed. Following rTMS, the neurophysiologic parameters were immediately re-assessed. The cap was removed and the participant was filmed again for blinded rating. Assessment for tolerability and safety were completed immediately after the session. The interval between intervention sessions was at least two days.

#### Primary outcome measure

The primary outcome measure was the change in the Toronto Western Spasmodic Torticollis Rating Scale (TWSTRS) severity subscore from baseline to after each of the rTMS interventions, including sham. The order of videos was randomized by random sorting and the blinded rater was not aware of the treatment assignments for the particular video.

#### rTMS

The principal investigator (SPR) and research coordinator enrolled subjects. The subjects were not told of their randomization assignment ordering. The sham was delivered with a double 70mm air cooled coil identical to the active coil, including giving an auditory signal with each pulse. Each subject underwent five rTMS sessions at least two days apart to complete the trial. The site order was randomized for each participant as described in Fig 1. The five sites were anterior cingulate cortex (ACC), dorsal premotor cortex (dPM), motor cortex (MC), sham over dPM (SHAM), and supplementary motor area (SMA) (See Table 1 for order of sites by participant). All sites were over the left hemisphere. For each session, rTMS was delivered at 0.2Hz at 85% of resting motor threshold (RMT) for 15 minutes (for a total of 180 pulses) using a Magstim Rapid^2^ stimulator (Magstim Co., Whitland, Dyfed, UK) with a double 70mm air cooled coil held with handle pointing backward and laterally at a 45-degree angle away from the midline.

#### dPMI

Surface gold electromyography (EMG) electrodes were placed on the right FDI muscle in a bipolar montage. The EMG signal was amplified using a conventional EMG machine (Nicolet Viking) with bandpass between 10 and 2000 Hz, digitized at a frequency of 5 kHz and fed into a computer for off-line analysis. The resting motor threshold (RMT) was determined over the primary motor cortex corresponding to the right (dominant) FDI. The coil over dPM was placed 2 cm anterior and 1 cm medial to the “hotspot” for FDI. With both coils placed, RMT and active motor threshold (AMT) during a 10% maximum voluntary contraction of FDI as measured by force transducer, were determined to the nearest 1% of stimulator output.

Two Magstim 200^2^ stimulators (Magstim Co., Whitland, Dyfed, UK), connected to two custom figure 8 coils with an inner loop diameter of 35 mm, were used. The motor cortex coil corresponding to the FDI “hotspot” was placed tangential to the scalp with handle pointing backward and laterally at a 45-degree angle away from the midline. The dPM coil was oriented to produce current in an anterior-to-posterior direction [10]. The coils overlapped slightly with the dPM coil contacting the scalp and the MC coil elevated. At least 24 motor evoked potentials (MEPs) (12 test pulses and 12 conditioning + test pulses delivered randomly) were collected from the right FDI at rest. The interstimulus interval (ISI) between the conditioning dPM TMS pulse and the test motor TMS pulse was 6 msec [11]. The intensity of the conditioning pulse was at 90% of AMT and the test intensity was 120% of RMT [11].

#### CSP

With the test pulse at 120% of RMT over the MC, at least 5 MEPs were collected from the right FDI at 10% maximum voluntary contraction. Off-line, the cortical silent period (CSP) durations were determined from the onset of the TMS test pulse artifact through the MEP to the resumption of EMG activity.

#### Safety and Tolerability

Participants were assessed immediately post-intervention for side effects or adverse events. Patients were asked to report tolerability of the study intervention on a 0 to 10 point scale where 0 represented completely tolerable and 10 represented completely intolerable.

### Statistical Analysis

The comparison of the primary outcome measure, the TWSTRS score before and after the interventions, and of the secondary outcome measures was calculated for each intervention separately. The change in scores was analyzed using the Kruskal-Wallis test, which is a non-parametric version of a one-way analysis of variance since the distributions were found not to be normal. To test the primary hypothesis that a particular stimulation site had greater effect than non-specific activation of the motor system, change in TWSTRS severity score pre- to post-intervention was analyzed by site. For the secondary outcomes (e.g. neurophysiology data), we paired CSP durations for pre-session 1 and pre-session 5 by paired t-test and similarly for percent dPMI (SAS version 9.3, Cary, NC, USA). *P* values less than 0.05 were considered as significant.

In a post-hoc analysis, the averaged TWSTRS severity score from Session 1 to Session 5 was compared to a historical control population using a one-sample Wilcoxon signed rank test given that the data were not normally distributed [14]. We compared this improvement in CD symptoms over time (indicated by lower TWSTRS severity score) to the natural history of waning botulinum toxin therapeutic effect from 8 week post-injection TWSTRS (corresponding to our Session 1 timing) to 12 week post-injection TWSTRS (corresponding to Session 5 timing). We used historical data from Comella, et al. (2011), which provides TWSTRS severity score data from these same corresponding time points (14.9 and 17.9, respectively) [14]. In addition, our study group had a mean TWSTRS severity score of 15.1 at the 8-week baseline, which is comparable to the Comella et al., 2011 group showing a TWSTRS score of 14.9 at 8 weeks—suggesting similar severity of CD in both studies [14]. *P* values less than 0.05 were considered as significant.

## Results

### TWSTRS Severity Score by Stimulation Site

**Fig 3:**
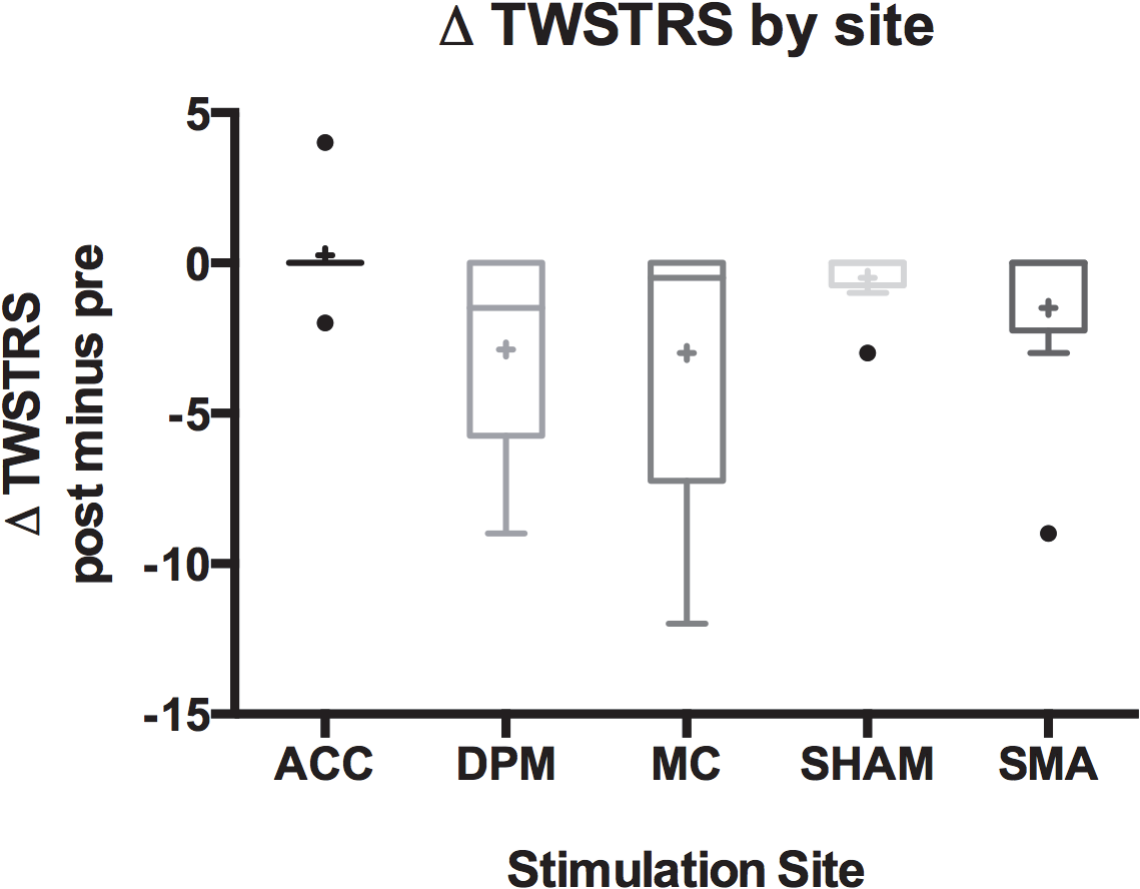
Boxplots of change in TWSTRS severity score by stimulation site showing greatest improvement over dPM and MC. A box in the boxplot represents first quartile to the third quartile with the median as the centerline. The outliers are represented by circles and the mean value as a cross. *Note that the ACC boxplot is collapsed since the first quartile is equal to the third quartile*.

The change in pre- to post-TWSTRS severity score was calculated for each site (with a positive change indicating worsening of symptoms and a negative change indicating improvement in symptoms) (n = 8). The mean change (± SD) in TWSTRS severity score by site was 0.25 ± 1.7 (ACC), -2.9 ± 3.4 (dPM), -3.0 ± 4.8 (MC), -0.5 ± 1.1 (SHAM), and -1.5 ± 3.2 (SMA). Boxplots are shown in Fig 3. All sites, except ACC, showed improvement in TWSTRS scores with the greatest improvement seen over dPM and MC. The pre-post change seen over dPM and MC were not significantly different (p>0.05). As the result was not significant, multiple comparisons were not performed and the p-values are presented unadjusted.

### TWSTRS Severity Score over time

**Fig 4:**
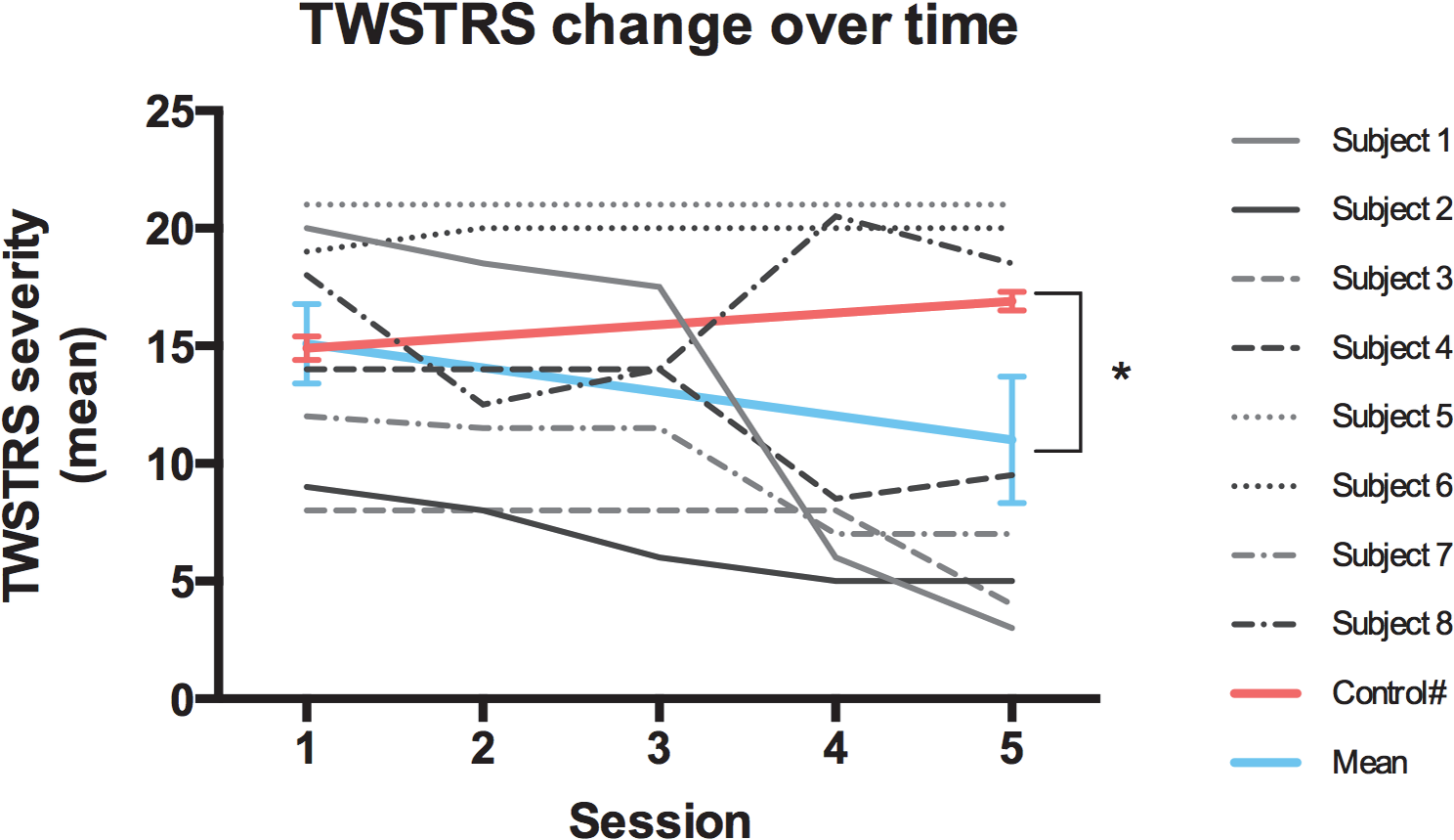
Change in TWSTRS severity score as a function of time shown from Session 1 to Session 5. Individual subject data shown as well as study group mean data shown in blue line. Red line represents historical control data from Comella et al., 2011 [14] showing similar severity scores at Week 8 post-botulinum toxin injection (study Session 1). *p = 0.01.

A one-sample Wilcoxon signed rank test of the averaged TWSTRS severity score from Session 1 to Session 5 compared to the historical control change from 8 weeks to 12 weeks-post injection revealed a significant difference between the improvement seen in our study compared to the wane of the clinical benefit of botulinum toxin over that 4 week interval (p = 0.008) (Fig 4).

### Neurophysiology Testing

**Fig 5:**
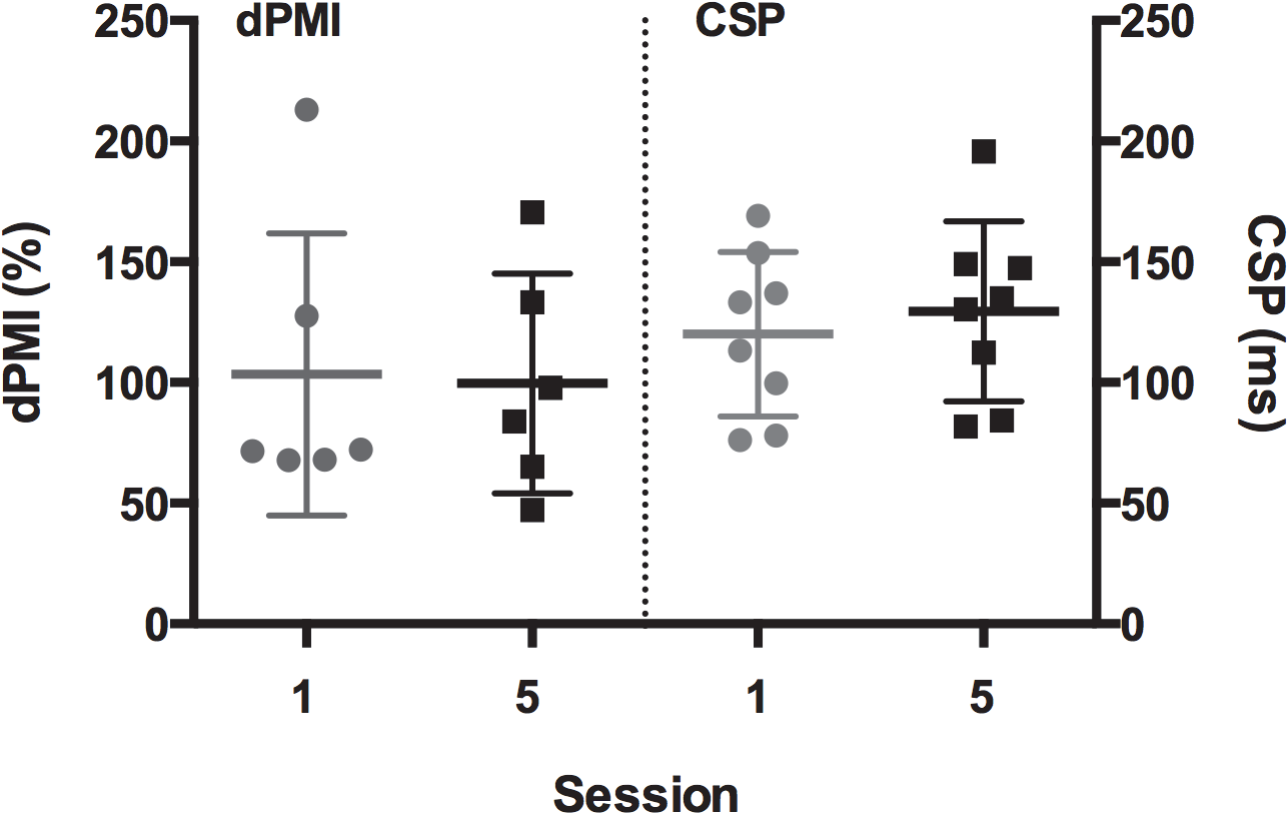
dPMI expressed as a percentage (MEP amplitude conditioned+test/MEP amplitude test alone *100) from pre-Session 1 and pre-Session 5. CSP durations shown in msec from pre-Session 1 and pre-Session 5.

dPMI is presented as a percentage of dPM conditioned motor evoked potential (MEP) amplitude to unconditioned (test) MEP amplitude (a percentage less than 100 indicates inhibition). We were able to record dPMI measures in 6 of the 8 subjects—in two subjects, the MEP amplitudes were <500μV even at maximum stimulator amplitude with the custom coils. Four of the six subjects exhibited enhanced dPMI (<100%) and two showed excitation (Fig 5). There was no difference in immediate pre- and post-intervention by session. There was also no cumulative effect over time seen when comparing pre-Session 1 dPMI (mean 103.4%, SD 58.4) to pre-Session 5 dPMI (mean 99.6%, SD 45.5) (Fig 5).

Similarly, CSP durations (in ms) did not change significantly in the immediate pre- to post-intervention interval nor when comparing durations from pre-Session 1 (mean 120.0 ms, SD 34.1) to pre-Session 5 (mean 129.5 ms, SD 37.3) (n = 8) (Fig 5).

### Safety and Tolerability

All participants completed all intervention sessions. There were no adverse events reported. Overall the interventions were tolerable to the participants (mean tolerability score 1.3, SD 1.6, range 0–4.8). There were no significant differences in tolerability by site: ACC (1.6, SD 2.8), dPM (1.5, SD 2.7), MC (1.6, SD 1.8), SHAM (0.8, SD 1.0), and SMA (0.9, SD 1.1).

## Discussion

rTMS over premotor and motor sites improved dystonia symptoms as measured by lower TWSTRS scores showing decreased severity. Both MC and dPM showed the largest change in the pre- to post-intervention TWSTRS rating. The stimulation was confined to the left hemisphere and showed benefit even though the neck muscles involved in CD can have both unilateral (e.g. trapezius) and bilateral (e.g. sternocleidomastoid) innervation [15]. This study provides proof-of-principle evidence that the same targets (MC and dPM) used in FHD are reasonable targets for further study in the CD population [4–8]. Moreover, all participants who began the interventions were able to complete all sessions and found it tolerable and safe.

Although encouraging, there are limitations to the interpretation of this study given the small sample size and these findings need to be replicated in a larger trial. In terms of reaching our target sites, we did attempt to target the ACC during this study, but it is possible that we were not able to reach this target with the figure-of-8 air cooled coil used in this study. Therefore we cannot draw a firm conclusion that ACC is not a potential therapeutic site, especially if an alternative coil was used for the targeting. Our choice of targets was based on previous studies that had been shown to be potentially therapeutic in focal dystonia; however, a recent case report described a new potential site, the left posterior parietal cortex, which was not examined in this study [16]. Remote targeting is another potential avenue of therapy to explore.

The trend, in one patient, to have a positive sham response is worth mentioning. One other subject had an even smaller (1 point in the TWSTRS score) to sham stimulation. Possible explanations for the sham response in these individuals include a placebo effect or a sustained benefit of sitting in a chair with their head touching the back of the chair during the rTMS session—a common sensory trick for many patients with cervical dystonia [17]. We also cannot exclude the possibility of a mild therapeutic effect as a result of the physiologic testing pre- and post-intervention affecting the sham condition. However, this was seen only in one subject and did not seem to have a significant effect on our findings.

In terms of the neurophysiologic data, we had previously shown in FHD patients [11] and recently in CD patients [12], enhanced dPMI compared to a healthy control population. In this current study four of our six subjects did show dPMI values < 100% as seen in our previous work. There was no significant effect change in dPMI over time. We did not power the study to detect change in these secondary outcomes and thus our ability to conclude that there was no effect on dPMI by the rTMS is limited. In addition, we have shown previously that patients with CD had shorter CSP than healthy controls suggestive of a loss of motor inhibition [12]. In our small population, the CSP at the pre-Session 5 was almost 10 msec longer than at the beginning of the trial but was not significantly different. Although these physiologic parameters did not seem to be effective surrogate markers in this exploratory trial, they may lend themselves to further examination in a larger study.

The most promising (and *unexpected*) finding in this study was the reduction in TWSTRS severity over time, suggesting a cumulative effect of rTMS sessions. Since the subjects were studied 8 weeks after the last botulinum injection, it is expected that the TWSTRS scores would worsen over time due to the waning of the effects of botulinum toxin. Seven of the eight subjects had maintenance or improvement in symptoms over the study duration. Only one subject approximated the natural history of therapeutic duration of botulinum toxin injection with gradual worsening in his TWSTRS score (although he showed immediate changes in TWSTRS score with several of the interventions administered) (Table 1). This yields support for a further exploratory, multisite Phase II trial to identify whether rTMS has the potential to step into the treatment gap seen with current therapy.

## Conclusions

This exploratory trial using low-frequency rTMS in CD showed immediate pre- and post-intervention improvements in TWSTRS severity—maximal over MC and dPM. TWSTRS severity scores were significantly lower at the end of the trial compared to the expected wane of botulinum toxin benefit during the treatment period. The intervention was tolerable and safe. These data support further study of this potential treatment in a larger population.

## Supporting Information

S1 CONSORT Checklist CONSORT Checklist. (DOC)

S1 Protocol Trial protocol. (DOC)
