# Supplementary material for: Repetitive Transcranial Magnetic Stimulation in Cervical Dystonia: Effect of Site and Repetition in a Randomized Pilot Trial
Source: PLoS One. 2015 Apr 29;10(4):e0124937. doi: 10.1371/journal.pone.0124937 (PMC4414555; doi:10.1371/journal.pone.0124937)
Supplement: S1 Protocol — (DOC) [file pone.0124937.s002.doc]

# Protocol:

1. Identification of optimal stimulation site for cervical dystonia symptoms: an exploratory study (Version 3.6.14)
2. IRB Review History

N/A

1. Objectives:

I am developing a research program that is centered on the investigation of transcranial magnetic stimulation (TMS) as a diagnostic and therapeutic agent for CD. TMS is an FDA-approved technique that involves generation of a brief magnetic field outside the skull that in turn can excite or inhibit brain tissue, muscle or nerve. In addition, when given repetitively, TMS can induce a temporary increase in inhibition (using low-frequency repetitive TMS (rTMS)) or an increase in excitation (using high-frequency rTMS). These changes can result in therapeutic effects.

The *overall objective* of this application is to target therapeutically the dysfunctional premotor-motor interaction in dystonia—and to provide a focused treatment of specific anatomical networks in order to reduce side effects and to improve symptom control over conventional therapies. We propose using rTMS over distinct premotor areas in patients with CD with the goal of improving symptoms and reducing unwanted side effects.

*Aim 1*: Identify the optimal stimulation site for transcranial magnetic stimulation therapy of symptoms of CD.

Based on our preliminary data, our working hypothesis is that repetitive TMS of a distinct premotor site (e.g. dorsal premotor cortex, ventral premotor cortex, supplementary motor area or anterior cingulate) will provide more effective treatment of CD than non-specific activation of the entire premotor region. Completion of this aim should lead to development of targeted TMS therapy for CD.

It is anticipated that this aim will yield a specific premotor anatomical target to be assessed in a Phase II clinical trial. The future direction is to resubmit the CDMRP grant using key data obtained from this pilot project. The CDMRP grant aims to address the question whether low-frequency rTMS can improve outcomes over those seen with botulinum toxin alone in the treatment of patients with cervical dystonia, specifically by a synergistic effect, to extend the duration of improvement in symptoms.

Successful completion of these studies should lead to rapid adoption of significantly more effective treatment of CD patients. Such results would be valuable for the large population of CD patients, but should also be generalizable to other large populations suffering from other focal dystonias.

1. Background:

After Parkinson disease and essential tremor, dystonia is the third most common movement disorder. It is characterized by abnormal posturing due to sustained muscle contractions, which not only leads to pain but also often causes *significant disability* in activities of daily living such as driving and reading. Primary focal dystonia such as cervical dystonia (CD) affects both genders and all ethnicities and races. The current gold standard treatment of botulinum toxin injections has limitations—painful, frequent injections as well as expected adverse events, which can include dysphagia, neck weakness, voice changes and fatigue. In addition, many CD patients do not have relief of their symptoms for the entire treatment period as the effect “wears off” prior to the next injection. Employment is affected by CD and in one study, a large percentage of patients were not employed even with standard treatment (Skogseid, 2005). *New therapies and new therapeutic targets are urgently needed in this disorder.* Previous work implies that CD is a “neurofunctional” disorder arising from abnormalities of neural connectivity or plasticity, rather than being caused by neurodegeneration. The functional rather than structural nature of the disorder raises the possibility that symptoms of CD can be persistently modified through noninvasive means.

Prior diagnosis and treatment studies have focused on the motor cortex, but our preliminary data suggests that other structures (including premotor cortex) may be more appropriate targets. Our preliminary data revealed abnormal inhibition from the dorsal premotor cortex to the motor cortex in patients with dystonia.

I have previously submitted an application for a clinical trial on rTMS combined with botulinum toxin and CD, supported by the federally funded Congressionally Defined Medical Research Program (CDMRP). The application received positive reviews but additional preliminary data supporting the specific premotor intervention site in CD patients was requested. The present application is designed to obtain this key preliminary data, to allow successful resubmission of the application. This investigation is novel in looking at the important contribution of the premotor cortex to both motor execution and motor planning abnormalities in focal dystonia.

1. Inclusion and Exclusion Criteria

Inclusion:

- Patients with idiopathic cervical dystonia
- Age 18 years or older
- Normal findings in the medical history, physical and neurological examination, except for dystonia
- Last treatment with botulinum toxin more than two months ago

Exclusion:

- History of seizure disorder
- Pregnancy- a pregnancy test will be performed for women of childbearing potential
- Symptoms of a clinically relevant illness in the 4 weeks before the first study day, including history of any other neurological disorders or conditions requiring the use of anti-depressants that are known to increase seizure threshold, neuroleptic medication, anticholinergic drugs and muscle relaxants with the exception of benzodiazepines
- History of neuroleptic medications/ prior use of neuroleptics
- Presence of pacemaker, implanted medical pump, metal plate or metal object in skull or eye

1. Number of Subjects (Recruitment Target)

Fifteen patients with CD meeting the eligibility requirements will be recruited and enrolled.

1. Recruitment Methods

Patients will be recruited from the Botulinum Toxin Clinic at the University of New Mexico Health Sciences Center, referrals from other neurologists that are sent “Dear Dr.” letters, and word of mouth from participants.

1. Study Timelines

Five study days will be scheduled with the patient. The time between study visits should be no less than 2 days (but can be longer than 2 days if necessary) to allow for “wash-out” of any treatment effects prior to the next intervention. The order of the interventions will be randomized. Subjects will be at least 2 months from previous botulinum toxin injection.

All enrollment activities will be concluded by July 1, 2014 with the analysis to be completed by the end of 2015.

1. Study Endpoints

Primary Study Endpoint

- The study will end once recruitment and completion of all study visits achieved.

Secondary Study Endpoint

- The study would end for an individual participant if they desired to withdraw from the study for any reason. We do not anticipate considerable discomfort to the participant during the study sessions, but if a participant found the experimental procedures intolerable, we would immediately stop the study for that participant.

1. Research Setting

For those participants that require an MRI, they will be scanned at the MIND Research Network. The five intervention appointments will all take place in the Noninvasive Neurostimulation Lab located at the CTSC.

1. Study Methods

This study is a randomized, sham-controlled, blinded-rater prospective study—based on the methodology of a successful pilot study in patients with blepharospam (Krantz, 2010). Each participant will receive 15-minute sessions of low-frequency (0.2 Hz) rTMS over the primary motor cortex (MC), dorsal premotor cortex (dPM), supplementary motor area (SMA), anterior cingulate cortex (AC), and a sham condition.

The primary outcome measure will be the change in the Toronto Western Spasmodic Torticollis Rating Scale (TWSTRS) score from baseline to after each of the rTMS interventions, including sham.

The patients will be videotaped and we will send the videos to our collaborator, Dr. Sule Tinaz at NIH, for blinded review of the TWSTRS score. The secondary outcome measures include: measurement of a physiologic marker of inhibition (PMI) pre- and post-intervention, subjective patient rating of symptoms, tolerability rating, and a side effect assessment. The rTMS interventions will be guided by a neuronavigation system (Brainsight™) to ensure consistent placement of coil (Figure 2). Brain MRI will be performed on subjects prior to the first study day for registration in the neuronavigation system.

Incentives: Participants will receive incentives on a graduated pay scale: Days 1-3, $20/day and Days 4-5, $40/day for a total of $140/subject who completed all 5 days plus $20/MRI scan time for a total of $160/subject.

Mileage Reimbursement: We will reimburse at the federal rate of $0.565/mile. The head accountant in the Neurology department will register the participants as vendors on Banner (document attached). This method requires obtaining their social security number, but only the accountant will see this information. The forms will be housed in the neurology department and the study coordinator will have no access to them. The "$600 rule" that is often brought up does not apply to mileage reimbursement as it is paying back money and is not perceived as a "benefit" of participating in the study. Therefore, a participant could theoretically be paid out $601+ for mileage and still not be reported to the IRS. There is a "60 day" rule, however. Any money reimbursed 60 days after the study date would be subject to tax. To avoid this issue, a check will be generated every thirty days, when necessary (although most study participants will be completed within 30 days time).

A paper listing of the link between subject number and identifiable personal information will be kept separate from the main coded dataset and secured in a locked cabinet. This link to the coded information will be kept intact until the end of the study period and in accordance with HRRC and federal requirements for clinical research.

1. List of Appendices

- Dear Dr./Pt letters
- Inclusion/Exclusion Criteria
- Tolerability scoresheet
- Toronto Western Spasmodic Torticollis Rating Scale (TWSTRS)

1. Data and Specimen Banking

- Information and clinical data collected as part of the study will be labeled with participant initials and a study number; information (without the name) will be entered into a computer database/locked file cabinet in the Principal Investigator's office.  The record linking the name to the MRN study ID number (which the MRI scan data is labeled with) will be kept indefinitely at the MRN in a confidential manner in case the participant needs future access to your MRI scan. Dr. Pirio Richardson and her study coordinator will have access to the study information. All other data will be stored for six years and then be destroyed.
- Identification information and data collected from each participant will be entered into the REDCap online data warehouse.
- For each study visit, there will be a neurological exam videotaped both pre- and post-intervention, totaling ten videos. These videos will include identifiable information (i.e. face and voice) and be sent via FedEx 2-day to the NIH collaborator.
- Participation in this study is completely voluntary. Each participant has the right to choose not to participate or to withdraw participation at any point in this study without affecting his/her health care or other services to which they are entitled.

1. Data Management

Data collection and storage

- Information and clinical data collected as part of the study will be labeled with the subject’s initials and a study number, which will be in a locked file cabinet in the locked Principal Investigator’s office; information will also be entered into a secure web-based computer database (REDCap).  The record linking your name to your MRN study ID number (which the MRI scan data is labeled with) will be kept indefinitely at the MRN in a confidential manner in case you need future access to your MRI scan. Dr. Pirio Richardson and her study coordinator will have access to your study information. All other data will be stored for six years and then be destroyed.

Digital recordings

- To participate in this study, agreeing to release the videos taken of the pre- and post-intervention scales is necessary. For each study visit, there will be a neurological exam videotaped both pre- and post-intervention, totaling ten videos. These videos will include identifiable information (i.e. face and voice).
- The videos will be uploaded to a password-protected computer and then deleted off the camera to ensure safety. For the blinded review, the videos will be burned onto a CD. Each file will be encrypted and must have a password to open. This password will be communicated to the collaborators via phone. The CD will be mailed to Dr. Tinaz using FedEx 2day with a required signature for delivery. The CD will be destroyed within one week of being viewed.

a. The password will be different for each CD.

b. We will communicate the password to the NIH collaborator via phone. The collaborator will write it down and destroy it after viewing the videos and completing the rating scales.

c. 7-Zip is a stronger level of security than Ecommerce uses[1] (i.e. Amazon). It is also universal, so it can be used on a MAC or PC interchangeably.

Statistical Plan

- In this exploratory study, the comparison of the primary outcome measure, the TWSTRS score before and after the interventions, and of the secondary outcome measures will be calculated for each intervention separately. The change in scores will be analyzed using repeated measures ANOVA. Locations will be compared with the Tukey adjustment for multiple comparisons. There are no prior data on the influence of rTMS patients with CD, and hence there is not way to calculate a sample size. We have estimated that 7 patients will give us enough information to find out which intervention over which area exerts the largest influence on cervical dystonia.

1. Provisions to Monitor the Data to Ensure the Safety of Subjects

- This study is not considered greater than minimal risk. The PI confers with her mentors on a regular basis in order to review safety precautions and any questions regarding the protocol.

1. Withdrawal of Subjects

- Any subject is free to withdraw from the study at any time.
- The investigator can remove a participant from the study at any time if she believes that by continuing is not in his/her best medical interest, or if he/she is unable to comply with the requirements of the study.

1. Risks to Subjects

- **TMS**: TMS is a safe procedure that has been used on many people to study the brain.  Most people do not find the stimulation painful, but occasionally strong contractions of scalp muscles can cause some discomfort of headache.  If you find the procedure too uncomfortable, you may discontinue it at any time.  Headaches usually go away promptly with nonprescription medication.  The noise of the TMS magnet may affect hearing, so you will be fitted with earplugs to wear during TMS.  Magnetic stimulation will not be performed in people who have pacemakers, implanted pumps or stimulators, or who have metal objects inside the eye or skull.  Please inform the investigators if you have any of these or known hearing loss.  This study employs paired pulse TMS, which is of equivalent safety to previous studies employing paired pulse TMS.  The risk of inducing a seizure with single, or paired-pulse, TMS is considered very low.  Seizures from single/paired-pulse TMS have only been reported in subjects with medically-intractable epilepsy very rarely (0.0-3%).  Safety studies using TMS in patients with neurological disorders have demonstrated no permanence.  Risks to a fetus are not known.  If you think you may be pregnant, you may choose not to participate in this study.
- **EMG**:  There is no risk associated with surface EMG.
- **MRI**: Radio and magnetic waves associated with MRI scans are not associated with any known adverse effects. MRI is non-invasive and considered minimal risk by the FDA. However, the scanner is a large magnet, so it could move objects with iron in them in the room during the scan. This means that loose metal objects, like coin currency or key chains, are not allowed in the MRI room. If you have a piece of metal in your body such as a pacemaker, nerve stimulator, piercings or certain metal surgical implants, you will not be allowed into the MRI room and cannot have an MRI. While in the scanner, you may be bothered by feelings of claustrophobia (fear of small spaces). If you feel uncomfortable (nervous or upset stomach) in the MRI scanner for any reason, tell the research staff. The MRI also makes loud ‘drum’ beating noises during the study. Headphones will be provided for your safety and comfort. There is a speaker in the MRI scan room as well as a window that allows the operator to view you during data collection. This allows the assistants to hear and see you at all times to ensure that you are comfortable and to allow them to respond if you are uncomfortable. You can stop the scan at any time.
- No long-term harmful effects from MRI are known. However, since the effect of MRI on early development of the fetus is unknown, subjects who are pregnant should not go in the MRI. If you are a woman 18 years of age or older and there is a possibility that you may be pregnant, you will be asked to take a urine pregnancy test before being allowed to participate in the study. Rarely, large or recent tattoos can heat up during an MRI scan and cause skin irritation like a sunburn, so the MRI technologist will want to see any tattoos you have prior to the scan.
- There are risks of stress, emotional distress, inconvenience and possible loss of privacy and confidentiality associated with participating in a research study. There may be other risks that are currently unknown. For more information about risks and side effects, ask the investigator.

1. Potential Benefits to Subjects

- There is the possibility of TMS directly improving symptoms related to cervical dystonia, or prolonging the benefit of botulinum toxin injections. This study will also yield generalizable knowledge about the subject’s disorder or condition and thereby improving the risk: benefit ratio in subsequent studies.
- The knowledge gained in this proposed research will identify a specific premotor area to be targeted with rTMS that is expected to result in a novel intervention that could enhance or replace current treatments for CD.  Adjunct treatments to botulinum toxin that could sustain treatment effect and decrease the frequency of re-injection could potentially result in cost savings without a decrease in symptom control. In addition to medical cost reduction, improved quality of life could be expected with the successful development of therapies that extend dystonia symptom control.

1. Vulnerable Populations

- This study is not targeting recruitment from vulnerable populations

1. Multi-Site Research

- This is a single-site study being completed at the University of New Mexico

1. Community-Based Participatory Research/Field Research

- N/A

1. Sharing of Results with Subjects/Incidental Findings

As all participants will be undergoing an MRI before participating in the study, they will receive a report from the MIND within a month after the scan. If there are any incidental findings then the PI will discuss what was found with the participant and offer him/her a copy of the scan on CD to keep in his/her permanent files.

1. Resources Available

The Principal Investigator

As PI in this project, I will be responsible for supervising and coordinating all aspects of the research. I am well prepared for this role. My fellowship at the National Institute of Neurological Disorders and Stroke in the Human Motor Control Section with Dr. Mark Hallett provided me with the experience and expertise to undertake clinical research with patients with a variety of movement disorders. During my fellowship at NINDS, I completed the NIH Clinical Center Clinical Research Curriculum Certificate with Commendation. I have also published multiple studies using transcranial magnetic stimulation in various movement disorders (e.g. dystonia, Parkinson disease and stroke) and well prepared to conduct the research in this population. In my capacity as Assistant Professor and Director of the Parkinson Disease and Movement Disorders Program at UNM, I see a wide-variety of patients with movement disorders and understand the diagnoses and treatments involved. I have been awarded a KL2 scholar position through the Clinical Translational Science Center at UNM. In this position, I have formal mentorship and training at achieving independent funding for my clinical research program. I have the expertise and experience to perform my role well in the proposed research.

Study Staff

Research Coordinator

The research coordinator will at all times be compliant with regulatory and training requirements for research staff. She is experienced at recruiting research subjects and ensuring their confidential participation. The PI will directly oversee the research coordinator.

Blinded rater

The blinded rater is a movement disorders trained neurologist with expertise in dystonia. The blinded rater will at all times be compliant with regulatory and training requirements for research staff.

Biostatistician

The biostatistician is an experienced statistician and compliant with regulatory and training requirements.

Mentors

The mentors for this project are internationally recognized experts in the fields of dystonia and transcranial magnetic stimulation. They are available for consultation and guidance if problems should arise.

Facilities

Noninvasive Neurostimulation Lab (located on 2^nd^ floor of CTSC)

This facility contains all the TMS, rTMS and Neuronavigation devices necessary for successful completion of the study. It is also a private location ensuring subject confidentiality.

MRI (at the Mind Research Network)

This is a well-known location for MRI research with easy parking and appropriate study staff in place.

1. Prior Approvals/Attachments Requiring Signatures

N/A

1. Confidentiality

The screening for ineligible candidates will be destroyed after the project's completion (12 months). The document containing contact/personal information for enrolled participants will be kept on a password-protected computer

It is imperative that video be taken for further analysis of therapeutic benefit after each of the 5 visit procedures. Dr. Sule Tinaz will also be performing a blinded review of the videos to confirm rating scale analysis.

Direct identifiers such as name, address and telephone number will be maintained in the event that subjects need to be re-contacted during the study duration due to re-consent or other event. The need to re-consent could occur if new information about safety parameters was learned during the duration of the study in a password-protected computer in the locked office of the principal investigator and kept for for six years under the HIPAA protocol.

The clinical data will be stripped of all identifying information, but the videos will contain head shots of the participants. This detail will be thoroughly covered during the consent process.

Additional details on confidentiality have been covered in Sections 13 and 14.

1. Provisions to Protect the Privacy of Subjects

All research appointments are conducted in the PI’s Noninvasive Neurostimulation Lab on the 2nd floor of the CTSC. The door is kept closed during experiment appointments.

1. Compensation for Research-Related Injury

If a participant is injured or becomes sick as a result of this study, UNMHSC will provide with emergency treatment, at the participant’s cost. No commitment is made by the University of New Mexico Health Sciences Center (UNMHSC) or MRN to provide free medical care or money for injuries to participants in this study.

In the event that a participant has an injury or illness that is caused by participation in this study, reimbursement for all related costs of care will be sought from the participant’s insurer, managed care plan, or other benefits program. If he/she do not have insurance, the participant may be responsible for these costs. He/she will also be responsible for any associated co-payments or deductibles required by the insurance company.

Contact information provided to all participants: the Human Research Review Committee (HRRC) at the University of New Mexico Health Sciences Center, Albuquerque, New Mexico 87131, (505) 272-1129.

1. Economic Burden to Subjects

N/A

1. Consent Process (including waiver request for HIPAA, waiver of HIPAA for recruitment only, Waiver of Informed Consent, and Alteration of Informed Consent)

Consent

- Please see accepted consent in CLICK

**HIPAA Authorization**

- Identifiers include videotaping the participants, which includes full-face coverage.
- Please see accepted HIPAA in CLICK.

**Non-English Speaking Subjects**

- No non-English speaking subjects are being recruited for this study.

**Planned Emergency Research Consents**

- N/A

**Cognitively Impaired Adults/ Adults Unable to Consent/ Use of a Legally Authorized Representative (LAR)**

- N/A

1. Drugs or Devices

**Medical Devices: Please respond to all questions in this section.**

A Magstim Rapid^2^ rTMS device and a Magstim BiStim paired-pulse TMS device are used in the study (The Magstim Company Limited, Spring Gardens, Whitland, Carmarthenshire, SA34 0HR, UK). The rTMS device is used to enhance inhibition over various areas in the premotor and motor cortex in order to improve symptoms of cervical dystonia.

Has an Investigational Device Exemption (IDE) application been submitted to the FDA?

No.

The device meets the requirements for an abbreviated IDE if all of the following are TRUE. Please articulate responses to all of the following:

- The device is not banned. **TRUE.** rTMS is FDA-approved for the treatment of treatment-resistant depression. In addition, TMS has been FDA-approved for the treatment of migraine.
- The device is not a significant risk device. **TRUE.** The UNM HRRC determined that the TMS devices are not significant risk devices according to this protocol.
- The sponsor or investigator will label the device in accordance with 21CFR812.5. **TRUE.** The devices are labeled accordingly.
- The sponsor or investigator will comply with the requirements of 21CFR812.46 with respect to monitoring investigations. **TRUE.** The investigator will comply with the requirements regarding monitoring of investigations.
- The sponsor or investigator will maintain research records required under 21CFR812.140 (b) and (5) and make the reports required under 21CFR812.150 (b) (1) through (3) and (5) through (10). **TRUE.** The investigator will maintain research records as described.
- The sponsor or investigator will ensure that participating investigators maintain the records required by 21CFR812.140 (a)(3)(i) and make the reports required under 21CFR812.150(a) (1) (2) (5) and (7). **TRUE.** The investigator will ensure that all study staff and investigators maintain records as described above.
- The sponsor or investigator will comply with the prohibitions in 21CFR812.7 against promotion and other practices. **TRUE.** The investigator will comply with all prohibitions against promotion and other practices.

Please complete the section below to determine if an IDE is required:

- Is the device FDA-approved for marketing and is it being used or investigated in accordance with its labeling.
  - **Yes.** rTMS and recently TMS are approved by the FDA for treatment of depression and migraine. Our proposal uses an even lower stimulation rate, suggesting continued use within clearly safe parameters.
- Is the device a diagnostic device?
  - No.
- Is the device undergoing consumer preference testing, testing of a modification, or testing of a combination of two or more devices in commercial distribution?
  - No.
- Is the device testing for the purpose of determining safety and effectiveness?
  - **Yes.** We are testing the effectiveness of rTMS in treatment of cervical dystonia symptoms.
- Is the device a custom device as defined in 21CFR812.3(b)?
  - No.

Provide a detailed description of device storage and accountability procedures:

- Where will the device be shipped within UNM?
  - The device is already housed at UNM in the Noninvasive Neurostimulation Lab.
- Where will the device be stored?
  - The device is stored in the locked Noninvasive Neurostimulation Lab at UNM on the 2^nd^ Floor of the CTSC.
- Who will have access to the device?
  - The access to the device will be limited to the PI who is fellowship trained in operating these devices and to the study personnel who are trained to use the devices safely.
- What security safeguards are in place to ensure proper accountability, access and storage of the device?
  - The devices are stored with the locked Noninvasive Neurostimulation Lab. The PI assumes accountability for the devices and their proper use.
- If the device is experimental, will it be labeled “Investigational Use Only”?
  - Yes.
- Who will be responsible for:
  - Device accountability. The PI.
  - Labeling/ dispensing. The PI.
  - State the qualifications of this person. The PI is fellowship trained in Human Motor Control and trained to use TMS devices. She has published in the areas of TMS and rTMS.

Describe how the investigational device is controlled, and how accidental use outside of approved research will be avoided.

The investigational device has two switches that must be pressed in sequence in order to turn on the device. The device is activated through computer control, which must be accessed through a password-protected computer and knowledge of the Signal software program. Finally, the handheld coils will not discharge unless 2 buttons on either side of the coil are held down simultaneously. The multistep procedure would ensure that an untrained person would be very unlikely to know how to operate the entire setup to cause discharge of the TMS. Again, the device is within a locked laboratory.

Non-Significant Risk Determination Section

- Is the device intended as an implant (≥30 days)?
  - No.
- Is the device for use supporting or sustaining human life?
  - No.
- Is the device for a use of substantial importance in diagnosing, curing, mitigating or treating disease or preventing impairment of human health?
  - No. This protocol is an exploratory study to determine whether rTMS has a beneficial effect on dystonia symptoms to provide preliminary data for a definitive clinical trial.
- Does the use of the device present a potential for serious risk to health, safety or welfare of the subjects?
  - The use of the device does not pose the potential for serious risk to health, safety or welfare of subjects. The protocol parameters are well within the bounds determined by the FDA for approval for the treatment of depression and migraine.

Explain why the use of the device in this study poses non-significant risk, and attach any other supporting information. In addition, explain whether the sponsor, the FDA or any other oversight organization has already made a risk determination for the device.

The device poses a non-significant risk to participants given that the parameters used are well within the FDA-approved rTMS therapy for the treatment of depression. The exclusion and inclusion criteria also ensure an added level of safety in using this device.
